# Developmental and reproductive performance of a specialist herbivore depend on seasonality of, and light conditions experienced by, the host plant

**DOI:** 10.1371/journal.pone.0190700

**Published:** 2018-01-05

**Authors:** Osariyekemwen O. Uyi, Costas Zachariades, Lelethu U. Heshula, Martin P. Hill

**Affiliations:** 1 Department of Zoology and Entomology, University of Fort Hare, Alice, South Africa; 2 Department of Animal and Environmental Biology, University of Benin, Benin City, Nigeria; 3 ARC–Plant Protection Research Institute, Cedara, South Africa; 4 School of Life Sciences, University of KwaZulu-Natal, Scottsville, South Africa; 5 Centre for Biological Control, Department of Zoology and Entomology, Rhodes University, Grahamstown, South Africa; Montana State University Bozeman, UNITED STATES

## Abstract

Host plant phenology (as influenced by seasonality) and light-mediated changes in the phenotypic and phytochemical properties of leaves have been hypothesised to equivocally influence insect herbivore performance. Here, we examined the effects of seasonality, through host plant phenology (late growth-season = autumn vs flowering-season = winter) and light environment (shade vs full-sun habitat) on the leaf characteristics of the invasive alien plant, *Chromolaena odorata*. In addition, the performance of a specialist folivore, *Pareuchaetes insulata*, feeding on leaves obtained from both shaded and full-sun habitats during autumn and winter, was evaluated over two generations. Foliar nitrogen and magnesium contents were generally higher in shaded plants with much higher levels during winter. Leaf water content was higher in shaded and in autumn plants. Total non-structural carbohydrate (TNC) and phosphorus contents did not differ as a function of season, but were higher in shaded foliage compared to full-sun leaves. Leaf toughness was noticeably higher on plants growing in full-sun during winter. With the exception of shaded leaves in autumn that supported the best performance [fastest development, heaviest pupal mass, and highest growth rate and Host Suitability Index (HSI) score], full-sun foliage in autumn surprisingly also supported an improved performance of the moth compared to shaded or full-sun leaves in winter. Our findings suggest that shaded and autumn foliage are nutritionally more suitable for the growth and reproduction of *P*. *insulata*. However, the heavier pupal mass, increased number of eggs and higher HSI score in individuals that fed on full-sun foliage in autumn compared to their counterparts that fed on shaded or full-sun foliage in winter suggest that full-sun foliage during autumn is also a suitable food source for larvae of the moth. In sum, our study demonstrates that seasonal and light-modulated changes in leaf characteristics can affect insect folivore performance in ways that are not linear.

## Introduction

Decades of studies have established the importance of host plant quality, including foliar nutrients, for herbivore performance (e.g. survival, growth and reproduction) and population dynamics [1–4]. Foliage quality is known to be influenced by a multiplicity of environmental factors such as seasonality (through its effects on host plant phenology) and light conditions [5–8]. From the viewpoint of nutritional ecology, the response of insect herbivores (in terms of survival, growth and reproduction) to independent and combined effects of seasonality and light conditions on the foliar quality of their host plants remains equivocal despite several studies [8–12]. Therefore, advancing our understanding of the relationship between foliage quality and phytophagous insect performance, and how abiotic conditions (e.g. seasonality, light conditions) singly, or in combination, influence this relationship is central to our knowledge of the nutritional ecology of insects and the evolution of their life histories.

The physiology, anatomy, phytochemistry of, and biochemical processes in individuals of the same plant species often vary according to host plant phenology due to seasonality. These variations are known to affect the nutritional quality of leaves by altering their physical and chemical characteristics such as leaf toughness, foliar nitrogen, water, phosphorus and carbohydrate contents as well as the concentrations of secondary chemicals [5–7, 9, 13]. For example, the concentration of foliar nitrogen, which is thought to be the most limiting macronutrient for insect herbivores, in trees and shrubs, is known to be higher during winter (and lower in autumn or summer) due to the plant’s biochemical response to low temperatures [13, 14]. Consequently, seasonal variation in foliar quality has been reported to influence the behaviour, performance, abundance and population density of insect herbivores [15, 16] and has been implicated (along with temperature) in insect population outbreaks [17].

In nature, individuals of the same species growing in different light environments (e.g. shaded vs full-sun habitats) often exhibit a degree of phenotypic and phytochemical variation [12, 18, 19] primarily because light intensity directly influences the physiological, growth and biochemical processes in plants by directly controlling carbon intake through photosynthesis [13, 20, 21]. The carbon/nutrient balance (C/NB) hypothesis [22, 23, 24] postulates that leaves of plants growing under sub-optimal levels of photosynthetically active radiation (e.g. in shaded habitat) should contain relatively more mineral nutrients, especially nitrogen and relatively less carbon-based secondary (defence) compounds (e.g. phenolics, tannins) compared to plants growing in full-sun environment. Leaves of plants growing in low light environments are often carbon limited and have been observed to exhibit reduced total non-structural carbohydrate (TNC) [12], increased foliar nitrogen [8, 18, 25, 26] and water content [8] as well as reduced tannins and phenolics [12], predicting that leaves of shaded plants should be better food for herbivorous insects. These light-mediated changes in the foliage quality of host plants have been demonstrated to influence insect herbivore performance in ways that are not straightforward [8, 12, 16, 18, 27], as evident in studies testing the C/NB hypothesis in this context [11, 28].

Despite reports that seasonality or plant phenology and light intensity alter plant quality and consequently indirectly affect insect performance, studies that explicitly or simultaneously assessed the relative roles of the indirect influence of both factors on host plant quality and on herbivores performance (through their effects on foliage quality) are scarce [6, 16]. If habitat heterogeneity due to sunlight conditions and temporal patterns of variation interact (e.g. some areas that are shaded in summer could become sunny in winter), leaves of individual plants of the same species in different habitats may be favourable for herbivores at different points in time. Such spatio-temporal variation would not only create temporally variable patterns of insect distribution and abundance [29], but may also influence the developmental and reproductive performance in phytophagous insects such as *Pareuchaetes insulata* (Walker) (Lepidoptera: Erebidae), a specialist folivore that was introduced from Florida, USA, into South Africa, for the biological control of the invasive alien plant, *Chromolaena odorata* (L.) King and Robinson (Asteraceae). Understanding how *P*. *insulata* responds to seasonal and spatial variations in the quality of its host plant will not only advance our knowledge of the nutritional ecology of this multivoltine moth, but may also help to explain its usually low population levels in the field in South Africa.

Previous studies, including ours, that evaluate the performance of insect herbivores on foliage from full-sun versus shaded environments, infrequently consider seasonality and host plant phenology or conduct such investigations at only one point in time [8, 18, 19]. The phenology of *C*. *odorata* is seasonal, with the flowering of mature plants in winter (between June and August) in the southern hemisphere. To our knowledge, the combined effects of seasonality (through its effects on host plant phenology) and light intensity on the leaf characteristics of *C*. *odorata* are unknown. Most importantly, it is not known whether the performance of the moth on shaded versus full-sun leaves varies according to season or host plant phenology. Here, we examined the effects of host plant phenology as influenced by seasonality (late growth-season = late summer/autumn and flowering-season = winter/early spring) and light conditions (shaded and full-sun) on the physical and chemical characteristics of *C*. *odorata* plants in order to determine the concentrations of the various potentially important nutrients in *C*. *odorata* plants in the different microhabitats and seasons. For purposes of convenience, the late summer/autumn trial will be called the ‘autumn trial’ and the winter/early spring trial will be called the ‘winter trial’ from here on. A further objective of this study was to determine whether seasonal and light-mediated changes in the phenotypic and phytochemical properties of *C*. *odorata* influence the performance of *P*. *insulata* by evaluating the survival, growth and reproductive metrics of the moth, by feeding the larvae on leaves obtained from both shaded and full-sun habitats during autumn and winter. We predicted that the physical and chemical characteristics of *C*. *odorata* leaves would be influenced by seasonality, and that the leaves of shaded plants would be of better quality for development and reproduction of *P*. *insulata* compared to full-sun ones, as proposed by the carbon/nutrient balance hypothesis. We also predicted that seasonal- and sunlight-mediated changes in the leaf characteristics of *C*. *odorata* plants will interactively influence the developmental and reproductive performance of *P*. *insulata*.

## Materials and methods

### Study organisms

*Chromolaena odorata* is perennial pioneering shrub that is native to the Americas (from southern USA to northern Argentina, including the Caribbean islands), but is now widespread in parts of Africa, Asia and Oceania, where it impacts negatively on agriculture, conservation of biodiversity, eco-tourism and livelihoods [30, 31]. The biotype of *C*. *odorata* that has invaded southern Africa, which originated from Cuba or Jamaica, is morphologically and genetically different from the more widespread biotype (Asia/West African biotype) invading Asia, Oceania, West, East and Central Africa [32]. Our study utilised the southern African biotype. In a high-light environment or in open situations, the shrub can grow up to 2–3 metres in height, but it can reach up to 5–10 metres when supported by other vegetation in semi-shaded habitats [30]. In South Africa, prolific flowering occurs during winter in June and July after the cessation of vegetative growth.

*Pareuchaetes insulata* is a multivoltine specialist herbivore that is native to the Americas (from southern USA to north-western South America), and was introduced into South Africa between 2001 and 2009 for the biological control of *C*. *odorata*. Under laboratory conditions and in field outbreak situations, the larvae of this moth can extensively defoliate *C*. *odorata*. The biology of this golden yellow moth is documented in Kluge and Caldwell [33] and Uyi et al. [34]. Following the release of over 1.9 million individuals of different life stages of *P*. *insulata* (from Florida—USA, Cuba and Jamaica), only the Floridian population (880,000 individuals of different life stages) is considered to have established after it was released at some 21 sites in KwaZulu-Natal (KZN) province, South Africa. It is unknown if populations from Cuba and Jamaica may have interbred with it and provided genetic material to the current population [35]. Although this insect did establish, its population levels are generally low in the field, albeit with occasional outbreaks and a degree of impact on the weed [36]. The variable performance and low population levels of the moth make it a difficult candidate to study in a field situation.

### Measurement of physical and chemical characteristics of leaves

Leaf characteristics of *C*. *odorata* plants growing in fields within the vicinity of the South African Sugarcane Research Institute (SASRI), Mount Edgecombe (29° 70’ S, 31° 05’ E), near Durban, South Africa, were studied. The South African Sugarcane Research Institute granted us permission to use their field sites and facilities. The field chosen consisted of full-sun (or open) and shaded habitats and measured 0.7 hectares. Three years (2010–2012) of annual weather data obtained from the South African Weather Service showed that the average daily temperature in the closest weather station [King Shaka International Airport (29° 36’ S, 31° 06’ E), Durban, South Africa] to the study site ranged between 11.2 and 28.9°C, and annual rainfall ranged between 870.5 and 1052.8 mm. The full-sun habitat was fully exposed to sunlight and was dominated by *C*. *odorata*, with a sparse population of *Lantana camara* L. (Verbenaceae), while the shaded habitat was partially exposed to sunlight and consisted of trees of *Syzygium guineense* (Wild.) DC. (Myrtaceae) and bugweed, *Solanum mauritianum* Scop. (Solanaceae). Light intensity (measured by a light meter, LX–101, Taiwan) differed significantly between the two habitats in autumn (mean ± SE: 1872.17 ± 23.36 and 347.21 ± 20.22 lux for full-sun and shaded habitat respectively; GLM ANOVA, *F*_1,19_ = 2436.82, *P* < 0.0001) and winter (mean ± SE: 1882.10 ± 34.251 and 358.23 ± 14.87 lux for full-sun and shaded habitat respectively; GLM ANOVA, *F*_1,19_ = 921.243, *P* < 0.0001) (see Uyi et al. [19] for further details about the study site). The leaf characteristics and insect performance study were conducted in winter/early spring 2013 (from July 15^th^ to October 6^th^, 2013 = winter trial) and late summer/autumn 2014 (from March 11^th^ to June 4^th^, 2014 = autumn trial) as a comparison, while the larval preference trial was conducted in early winter 2017 (6-8^th^ June 2017). All plants used in the winter trials were at the flowering and seeding stage whereas the ones used in the autumn trials were nearing the end of their vegetative growth season, and had not started flowering. It is possible that there might be a difference in the chemistry / nutrient allocation of flowering versus seeding plants. In *C*. *odorata*, the initiation of flowering in winter causes all further production of new leaves to cease. One of the reasons why we chose late autumn plants as opposed to spring plants plants for this study is because all occasional outbreaks of the moth usually occur during this period [36].

The specific leaf weight (SLW), which provides a physiological estimate of ‘leaf toughness’ [37] of 100 fully expanded leaves (taken from the upper half of the plants) obtained from 20 plants per habitat type (5 leaves per plant) in August 2013 (winter) and April 2014 (autumn) was estimated in both shaded and full-sun habitats following the methods described in our earlier study [19]. Different 20 plants were sampled in August 2013 and April 2014. To determine whether seasonality and habitat conditions influence the foliar chemistry of *C*. *odorata* plants, leaf materials collected from 10 randomly selected *C*. *odorata* plants along a 20 m transect in each habitat in both autumn and winter were subjected to analyses at the laboratories of the Fertilizer Advisory Service, SASRI and KZN Department of Agriculture and Rural Development, South Africa. Following measurement of the wet weight of the leaf materials, they were dried for 72 h at 65°C and N and C contents were determined as a percentage of dry weight using a TruSpec^®^ CN analyser (LECO, St Joseph, MI, USA). After ashing of a subsample, P was determined colorimetrically [38], and K, Ca, and Mg using atomic absorption spectrophotometry. The amount of the total non-structural carbohydrate (TNC) in leaves was analysed using the acid hydrolysis procedure [39]. Finally, the acid detergent lignin content was analysed using the methods described in van Soest [40], while water content (%) was calculated using the formula: [(leaf fresh weight–leaf dry weight) / leaf fresh weight] × 100%.

### Development and reproductive performance of *Pareuchaetes insulata*

For the winter trial, the larvae used in the insect performance experiments were obtained from eggs laid by *F*_*1*_ adult females whose original parents were collected in May 2013 on light traps at the Sappi Cannonbrae plantation, Umkomaas (30^o^ 13’ S, 30^o^ 46’ E) (south coast of KZN province), South Africa. For the autumn trial, the larvae used in the insect performance experiments were obtained from eggs laid by *F*_*1*_ adult females whose original parents were collected in February 2014 on light traps at the same location as above. The parents (males and females) were placed in aerated 700 ml plastic containers, each with a 5 cm diameter mesh window at the top, with *C*. *odorata* stem cuttings plugged into a moistened Oasis^TM^ floral foam block (5 × 5 × 3 cm) wrapped with aluminium foil for egg laying. They were provided with a cotton-wool ball soaked with a 50% (wt/vol) honey solution and kept in the laboratory (25 ± 2 ^o^C, 65 ± 10% relative humidity (RH), L12:D12) at the Weeds Biocontrol Research Laboratory of the ARC-Plant Protection Research Institute (ARC-PPRI), KZN, South Africa (29° 32’ S, 30° 16’ E). Hatched larvae (from eggs laid) were fed on leaf cuttings obtained from plants in 25 cm-diameter pots (see Uyi et al. [41] for details of the potting medium). The resulting adults (1 virgin female and 2 newly eclosed males) were placed in 700 ml containers as described above. The eggs laid by these females were used for this study.

The performance experiments were conducted in a temperature-controlled room maintained at 25°C, 68% RH and 12D:12L photoperiod at SASRI. Hygrochron iButtons (model DS 1923, Maxim Integrated Products, San José, USA, 0.5°C accuracy) were used to measure temperature and RH at hourly intervals during winter (temperature range, 23.69 to 26.19 ^o^C; mean ± SE, 24.84 ± 0.01 ^o^C; RH range, 66.5 to 75.3%; mean ± SE, 70.51 ± 2.13%) and autumn (temperature range, 24.45 to 26.74 ^o^C; mean ± SE, 24.95 ± 0.01 ^o^C; RH range, 68.4 to 78.1%; mean ± SE, 71.34 ± 2.16%). For both the winter and autumn study, newly hatched (unfed) *F*_*1*_ larvae were placed individually, using a fine brush, into transparent 100 ml aerated plastic containers (one larva per container), each with a circular screen window of 2.5 cm diameter at the top, lined at the bottom with moistened filter paper to maintain RH, and fed on *C*. *odorata* foliage (fully expanded leaves taken from the upper half of plants in the field) obtained from either shaded or full-sun habitat. All leaf materials were obtained fresh from over eight plants per habitat on each collection date (every 48 h) at the field site and replaced by new materials from different plants every 48 h. One hundred replicates (= 100 larvae) were used for each foliage type. In this way, a total of 400 individuals (100 per foliage type in autumn and winter trials) of *P*. *insulata* were studied for two successive generations. Therefore 800 larvae were studied. The autumn and winter trials were conducted using Completely Randomized Design (CRD). Leaf materials were replaced every 48 hours and frass was removed at same interval for hygienic reasons [42]. The larvae were monitored daily until pupation and/or adult eclosion in order to record mortality and follow the duration of larval instars and the pupal stage. The following variables were measured or recorded for both shaded and full-sun foliage-fed individuals in both seasons: (1) total immature development time (duration from egg hatch to adult eclosion), (2) pupal mass, and (3) growth rate [pupal mass (mg) / development time]. We also calculated Maw’s host suitability index (HSI) using the following equation: HSI = (female pupal mass × % pupation) / immature development time (for rationale, see Maw [43]). Newly hatched larvae (*F*_*2*_ or their progeny) resulting from *F*_*1*_ adults in this experiment were also subjected to similar treatment as described above (for the larval performance trial). Two generations of the insect were studied, because only two generations of this insect can be obtained within a particular season.

When the adults of both the “parental generation” (*F*_*1*_) and progeny (*F*_*2*_) eclosed, 1 virgin female and 2 newly eclosed males that had fed on either shaded or full-sun foliage as larvae were placed in 700 ml containers as described above (as was done for oviposition of field-collected adults) but they were provided with stem cuttings (with leaves) of the plant type (shaded and full sun) they had fed on as larvae. Previous studies suggest that night-active species do not discriminate (for oviposition) between foliage in the sun or shade as the proximal light micro-environment encountered by the ovipositing females do not differ at night [44]. Therefore, we maintained all experimental containers under the same light condition, (L12:D12). During the winter trials, 43 replicates each were used for full-sun and shaded habitat trials (86 in total), while during the autumn trials, 34 and 35 replicates (69 in total) were respectively used for full-sun and shaded habitat trials. The containers and leaves were examined daily to record the following: (i) adult longevity and (ii) numbers of eggs laid.

### Larval preference test

To test the attractiveness of full-sun and shaded foliage of *C*. *odorata* to *P*. *insulata*, freshly collected young, fully expanded leaves of each of these two foliage types were used following the methods described in Uyi et al. [41]. Rectangular pieces of leaf tissue (20 × 40 mm) were placed in 140-mm-diameter Petri dishes containing a 90-mm-diameter disc of filter paper moistened with 0.6 ml of water. Two rectangles of leaf tissue from shaded *C*. *odorata* foliage were placed alternately around the edge of the Petri dish with those of full-sun and 20 unfed neonate *P*. *insulata* were placed haphazardly (but not on the leaves) using a fine brush in each Petri dish. Twenty replicates were used per foliage type and the leaf tissues used were taken from 40 different plants. Following a 24 hour treatment, each dish was opened and the number of larvae on each leaf piece was counted.

### Statistical analysis

The effect of season (plant phenology: late growth-season vs flowering-season plants) and habitat condition on leaf characteristics were evaluated using univariate General Linear Model analysis of variance (GLM ANOVA). When the overall results were significant in a two-way analysis, the differences among the treatments were compared using the Tukey’s HSD test because of the equality of sample sizes. We performed statistical analyses based only on the number of individual larvae that successfully eclosed as adults. Data from pupae that failed to eclosed were discarded because we wanted to be sure that handling (disturbance) during measurement (= weighing) did not negatively affect adult eclosion success. Data from two successive generations per habitat type within each season were combined and used for the analyses of insect performance metrics to allow for a more robust analysis. Pearson’s χ^2^ test was used to compare the effect of season and habitat condition on total immature survival. The effect of season (autumn vs winter) and habitat condition (shade vs full-sun) on total development duration, pupal mass, growth rate, host suitability index (HSI), number of eggs laid and adult longevity were evaluated using GLM ANOVA. When the overall results were significant, the differences among the treatments were compared using Tukey-Kramer’s test. The percentage preference for foliage type by neonate larvae was evaluated using student’s *t* test With the exception of the GLM ANOVA that was performed using SPSS Statistical software, version 16.0 (SPSS, Chicago, USA), all other analysis were performed using Genstat 14.0 (VSN International, Hemel Hempstead, UK).

## Results

### Leaf characteristics

**Fig 1:**
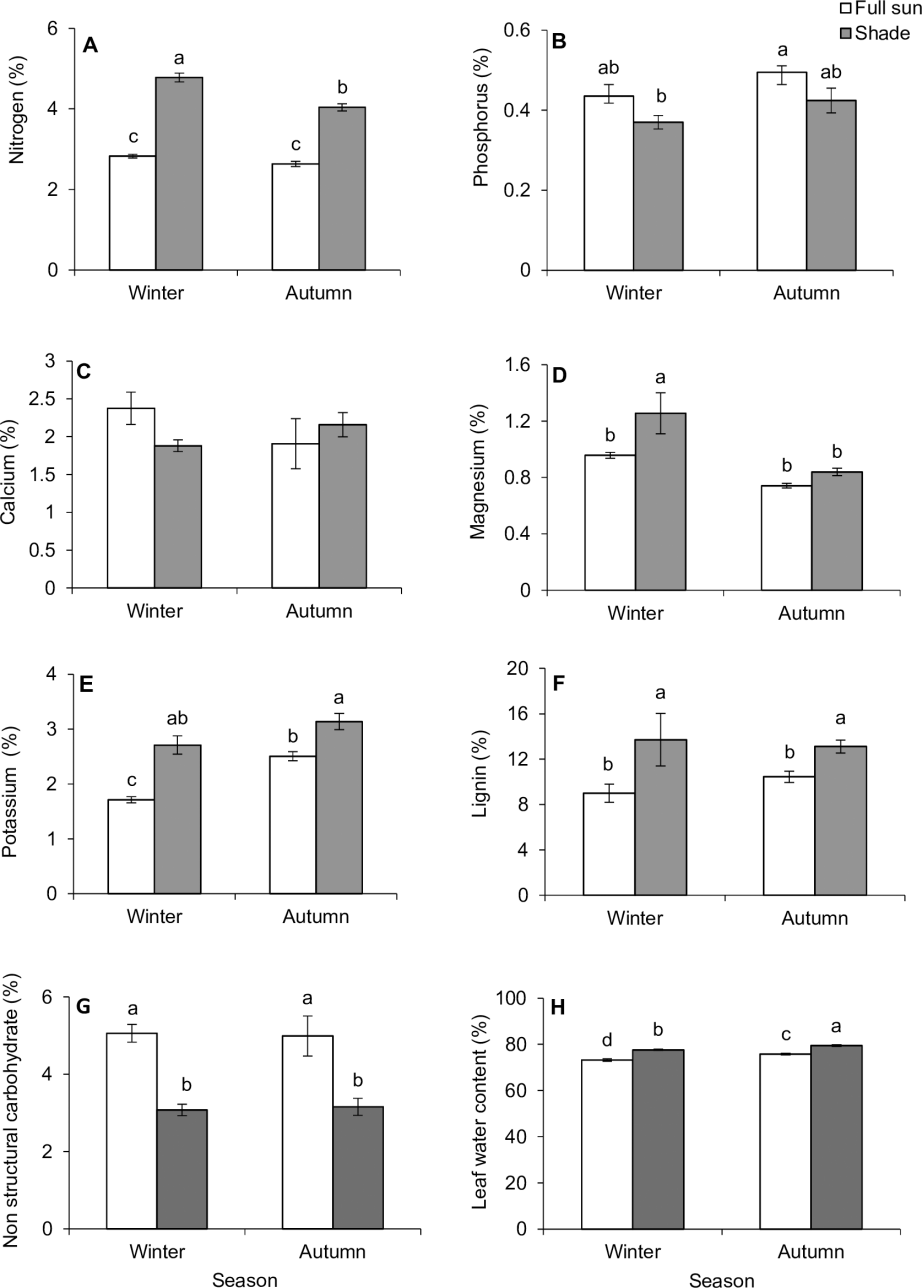
Seasonal (autumn vs winter) variation in the phytochemistry (including nutrients, % dry mass) of the leaves of *Chromolaena odorata* plants in two habitats (shade vs full sun). Data represent means ± SE. Bars within each graph not sharing a common letter differ significantly (*P* < 0.05) after Tukey’s Honest Significant Difference (HSD) test.

**Fig 2:**
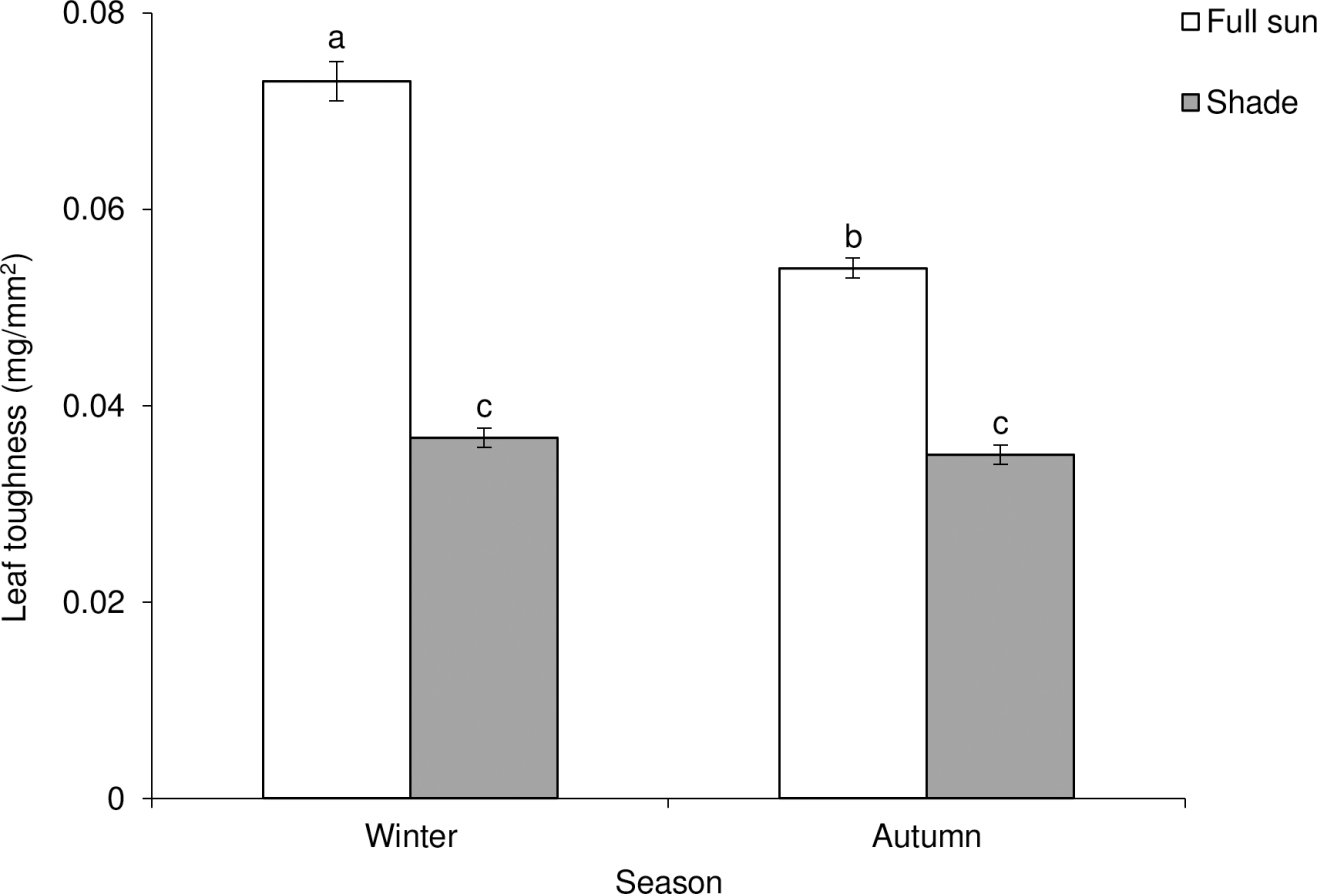
Seasonal (autumn vs winter) variation in leaf toughness [as indicated by specific leaf weight (SLW)] of *Chromolaena odorata* plants in two habitats (shade vs full sun). Data represent means ± SE. Bars within each graph not sharing a common letter differ significantly (*P* < 0.05) after Tukey’s Honest Significant Difference (HSD) test.

Results indicated that seasonality and habitat conditions independently and interactively impacted foliar nitrogen content of *C*. *odorata* plants. Foliar nitrogen concentration was 69.2% and 52.5% higher in shaded plants (relative to full-sun plants) for winter and autumn, respectively (Table 1; Fig 1A). Foliar nitrogen concentration was 18.6% higher in winter compared to autumn in the shaded habitat, but its concentration in the full-sun habitat did not differ between winter and autumn plants. Phosphorus concentration was slightly higher in the full-sun habitat in both autumn and winter (Table 1; Fig 1B). The calcium concentration did not differ between seasons or between habitats (Table 1; Fig 1C). Although magnesium concentration was higher in shaded habitat in winter, it did not differ between habitats in autumn (Table 1; Fig 1D). The concentration of potassium was higher in autumn than winter and the shaded habitat had significantly higher concentration compared to full-sun habitat (Table 1; Fig 1E). Lignin content did not differ between seasons, but was statistically higher in shaded habitats in both autumn (13.11%) and winter (13.72%) (Table 1; Fig 1F). Although the concentration of total non-structural carbohydrate (TNC) did not vary between autumn and winter, it was 64.3 and 50.0% higher in the full-sun habitat in winter and autumn respectively (Table 1; Fig 1G) compared to the shaded habitat. Leaf water content was higher for plants growing in the shaded habitat in both autumn and winter and was higher in autumn compared to winter (Table 1, Fig 1H). Specific leaf weight (SLW, an indication of leaf toughness) was influenced by seasonality (or host plant phenology), habitat condition and their interaction (Table 1, Fig 2). Leaf toughness in the full-sun habitat was 97.35% greater than that in the shaded plants in winter and 54.28% greater than shaded habitat in autumn (Table 1, Fig 2).

**Table 1:** Statistical details (GLM ANOVA) for the analyses of the effects of season (autumn vs winter) and habitat condition (shade vs full-sun) on physical and chemical characteristics of *Chromolaena odorata* leaves.

**Analysis**	**Source of variation**	**DF**	**MS**	**F-value**	*P- value*
Nitrogen	Season	1	2.162	33.58	**< 0.0001**
	Habitat	1	28.190	437.86	**< 0.0001**
	Season × Habitat	1	0.761	16.25	**< 0.0001**
	Total	39			
Phosphorus	Season	1	0.032	5.35	**0.027**
	Habitat	1	0.046	7.61	**0.009**
	Season × Habitat	1	0.001	0.01	0.904
	Total	39			
Calcium	Season	1	0.091	0.47	0.499
	Habitat	1	0.150	0.77	0.387
	Season × Habitat	1	1.315	7.13	0.071
	Total	39			
Magnesium	Season	1	0.998	17.78	**< 0.0001**
	Habitat	1	0.392	6.98	**< 0.0001**
	Season × Habitat	1	0.101	1.78	0.19
	Total	39			
Potassium	Season	1	3.733	24.66	**< 0.0001**
	Habitat	1	6.609	43.67	**< 0.0001**
	Season × Habitat	1	0.334	2.21	0.146
	Total	39			
Lignin	Season	1	1.600	0.10	0.757
	Habitat	1	138.681	8.43	**0.006**
	Season × Habitat	1	10.283	0.63	0.434
	Total	39			
Non-structural	Season	1	0.000	0.00	0.982
Carbohydrate	Habitat	1	36.243	36.88	**< 0.0001**
	Season × Habitat	1	0.052	0.05	0.819
	Total	39			
Leaf water content	Season	1	47.524	25.68	**< 0.0001**
	Habitat	1	165.345	89.34	**< 0.0001**
	Season × Habitat	1	1.024	0.55	0.462
	Total	39			
SLW(Leaf toughness)	Season	1	0.012	43.51	**< 0.0001**
	Habitat	1	0.079	273.99	**< 0.0001**
	Season × Habitat	1	0.008	28.07	**< 0.0001**
	Total	399			

DF: degrees of freedom; MS: mean squares.

Statistically significant values are indicated in bold.

### Developmental and reproductive performance of *Pareuchaetes insulata*

**Fig 3:**
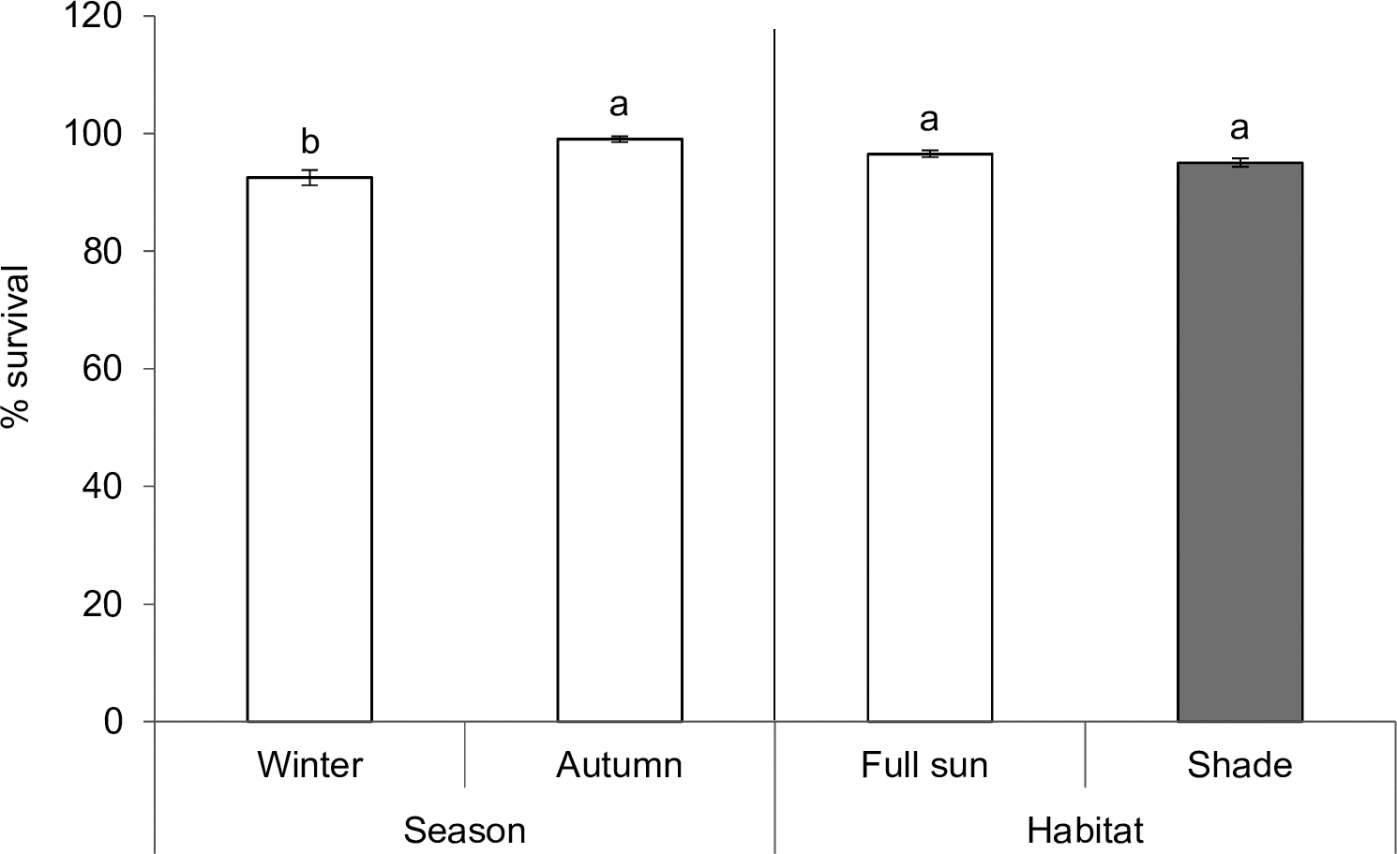
Seasonal (autumn vs winter) variation in percentage survival (mean ± SE) of combined immature stages of *Pareuchaetes insulata* reared on *Chromolaena odorata* leaves from two different habitats (shade vs full sun). Bars with different letters are significantly different (Pearson *χ*^*2*^; *P* < 0.05). Each bar represents percentage survival out of 400 first instar larvae monitored until adult eclosion.

**Fig 4:**
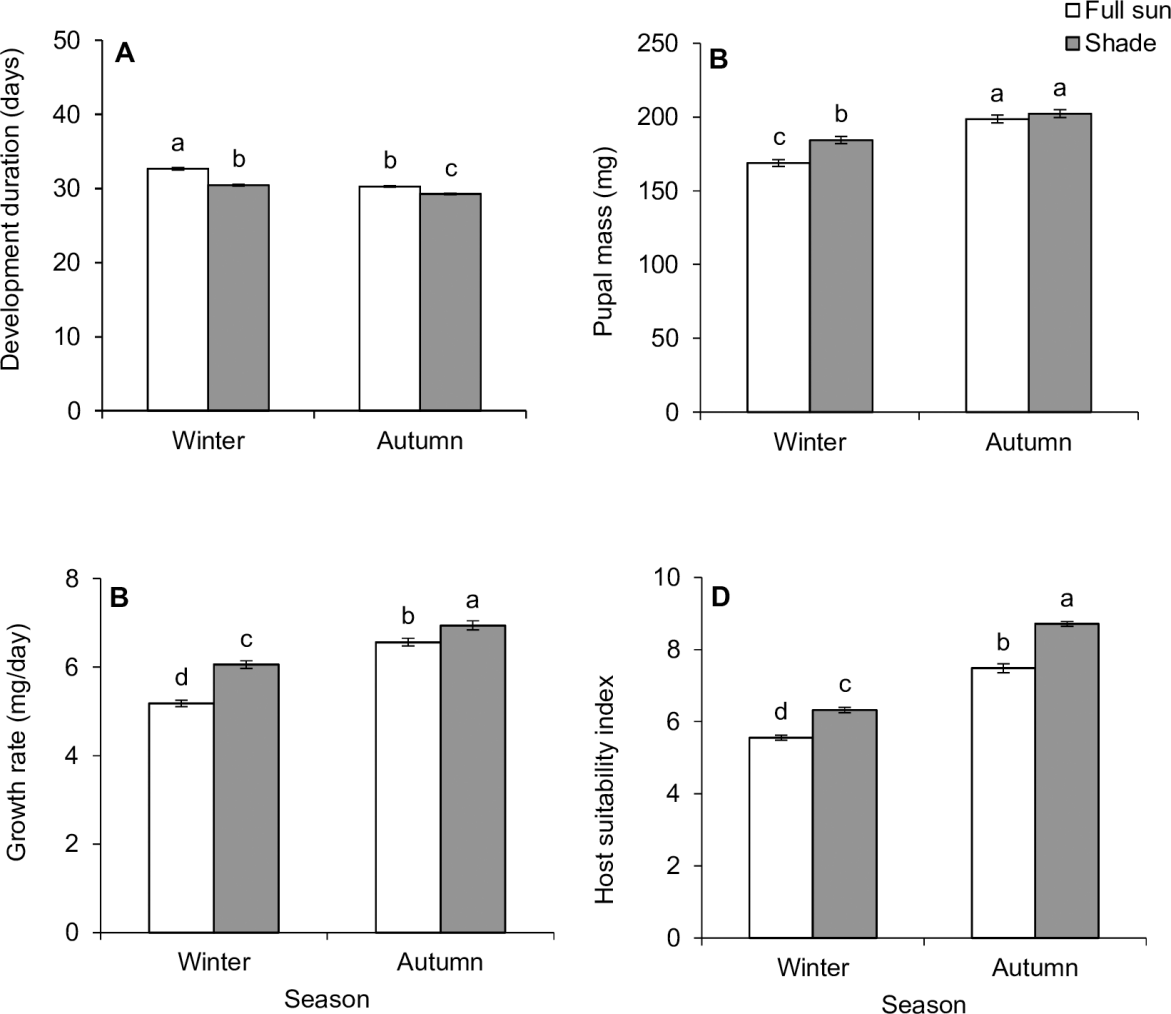
**Seasonal (autumn vs winter) variation in total development duration (a), pupal mass (b) growth rate (c) and host suitability index (d) of *Pareuchaetes insulata* reared on *Chromolaena odorata* leaves from two different habitats.** Data represent means ± SE. Bars within each graph not sharing a common letter differ significantly (*P* < 0.05) after Tukey-Kramer test.

**Fig 5:**
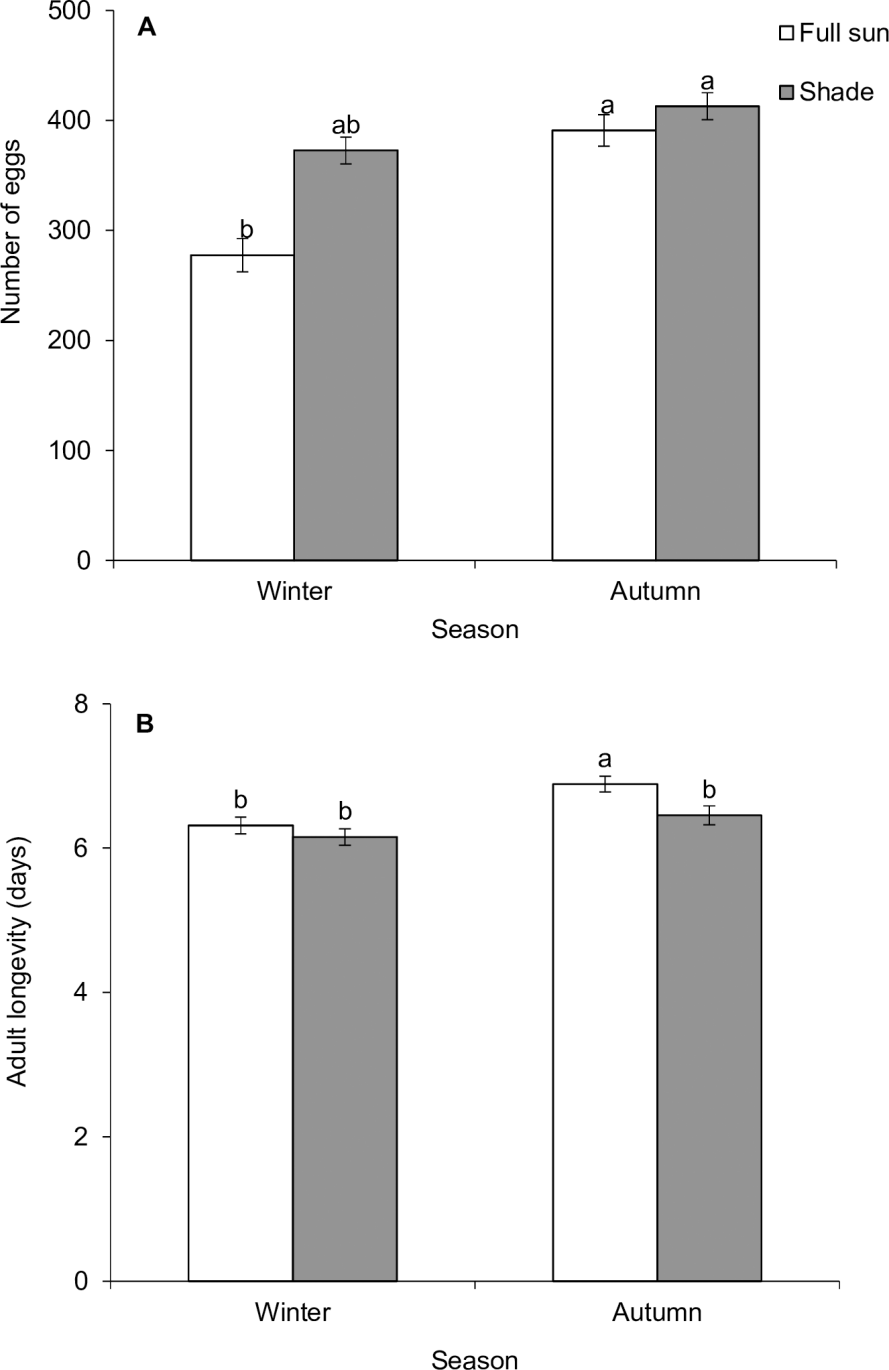
**Seasonal (autumn vs winter) variation in mean number of eggs (a) and mean adult longevity (b) of *Pareuchaetes insulata* reared on *Chromolaena odorata* leaves from two different habitats (shade vs full sun)**. Data represent means ± SE. Bars within each graph not sharing a common letter differ significantly (*P* < 0.05) after Tukey-Kramer test.

Overall, immature survival of *P*. *insulata* (first instar to adult eclosion) was influenced by seasonality (Pearson χ^2^ = 5.17, d.f. = 1, P = 0.023), with autumn foliage supporting higher survival compared to winter foliage (Fig 3). However, no significant difference in immature survival due to foliage types was detected (Pearson χ^2^ = 0.28, d.f. = 1, P = 0.599) (Fig 3). Total development time, pupal mass, growth rate, host suitability index (HSI) and fecundity were influenced by seasonality (or host plant phenology), habitat condition, and their interactions (Table 2; Figs 4A–4D and 5A). Total development was significantly faster on shaded leaves in both autumn and winter (Fig 4A). Although faster development time was evident in autumn compared to winter irrespective of habitats, it was similar between individuals that fed on shaded leaves in winter and those that fed on full-sun leaves in autumn. Generally, autumn foliage supported heavier pupal mass compared to winter ones (Fig 4B). Pupal mass was evidently heavier in individuals that fed on shaded leaves in winter compared to those that fed on full-sun foliage in the same season, but this variable did not differ between foliage types in autumn (Fig 4B). Individuals reared on autumn foliage had a higher growth rate than those that were fed on winter foliage (Fig 4C). Irrespective of season or host plant phenology, growth rate was always higher in individuals fed on shaded leaves compared to those that fed on full-sun leaves (Fig 4C). Larvae fed on shaded or autumn leaves had higher Maw’s HSI values (Fig 4D).

**Table 2:** Statistical details (GLM ANOVA) for the analyses of the effects of season (autumn vs winter) and habitat condition (larval food source) on total development duration, pupal mass, growth rate, host suitability index, number of eggs and adult longevity.

**Analysis**	**Source of variation**	**DF**	**MS**	**F-value**	*P- value*
Total development	Season	1	622.166	199.17	**< 0.0001**
Duration	Habitat	1	483.049	154.65	**< 0.0001**
	Season × Habitat	1	50.760	16.25	**< 0.0001**
	Total	765			
Pupal mass	Season	1	110934.110	94.43	**< 0.0001**
	Habitat	1	16938.000	14.42	**< 0.0001**
	Season × Habitat	1	6848.231	5.83	**0.016**
	Total	765			
Growth rate	Season	1	250.196	171.68	**< 0.0001**
	Habitat	1	73.726	50.59	**< 0.0001**
	Season × Habitat	1	11.962	8.21	**0.004**
	Total	765			
Host suitability index	Season	1	385.161	547.03	**< 0.0001**
	Habitat	1	84.327	119.77	**< 0.0001**
	Season × Habitat	1	5.030	7.14	**0.008**
	Total	356			
Number of eggs	Season	1	226299.110	30.96	**< 0.0001**
	Habitat	1	152326.400	20.84	**< 0.0001**
	Season × Habitat	1	51417.201	7.03	**0.009**
	Total	154			
Longevity	Season	1	36.459	13.93	**< 0.0001**
	Habitat	1	17.443	6.69	**0.01**
	Season × Habitat	1	3.611	1.38	0.24
	Total	765			

DF: degrees of freedom; MS: mean squares.

Statistically significant values are indicated in bold.

Generally, autumn foliage supported higher number of eggs compared to winter foliage (Fig 5A). Number of eggs was evidently higher in females that fed on shaded leaves (as larvae) in winter compared to those that fed on full-sun foliage in the same season, but this variable did not vary between foliage types in autumn. Adult longevity did not differ as a function of foliage type in winter, but varied between habitats in autumn–with individuals that fed on full-sun foliage living slightly longer than their counterparts that were reared on shaded leaves (Table 2, Fig 5B). Interestingly, individuals that fed on full-sun foliage in autumn had heavier pupal mass, increased number of eggs and higher HSI score compared to their counterparts that fed on shaded or full-sun foliage in winter.

### Larval preference

Neonate larvae (newly emerged larvae) of *P*. *insulata* did not display any distinguishable preference for, or attractiveness to either shaded or full-sun foliage (mean ± SE, full-sun: 51.33 ± 3.09; shade: 48.67 ± 3.07; t_1_,_38_ = 0.61, P = 0.546).

## Discussion

The higher concentration of foliar nitrogen in shaded plants in winter has not been previously reported in *C*. *odorata*, however, it has been shown in several plant species that foliar nitrogen increases in winter compared to summer or autumn [13, 45, 46]. The seasonal variation in leaf nitrogen suggests that the plants alter their nitrogen concentration to maximize daily nitrogen-use efficiency of carbon gain in response to varying seasonal temperatures, as has been reported by other workers [13]. The lower leaf water content and the increased leaf toughness in winter (relative to autumn) might be due to the seasonally dry winter (low winter rainfall) in areas invaded by *C*. *odorata* in South Africa. The presence of significant interactions in foliar nitrogen content and leaf toughness between light conditions and seasonality suggests that the effect of light condition was more apparent or stronger in winter compared to autumn. As predicted by the carbon nutrient balance (C/NB) hypothesis [22, 23, 24], leaves of *C*. *odorata* plants growing under sub-optimal levels of photosynthetically active radiation (= shaded plants) had relatively more mineral nutrients (e.g. increased nitrogen, magnesium, potassium and water contents) and reduced leaf toughness. The reduced leaf nitrogen and water as well as the increased concentration of TNC and leaf toughness in leaves of plants growing in full-sun concurs with the findings for other plant species [12, 18, 47], although a few studies have reported the opposite pattern [27]. The ostensibly higher lignin content in shaded habitat in both seasons might seem incongruous, however, it is consistent with the findings of Onoda et al. [48] who found that shaded leaves also had higher acid detergent fibre (ADF, such as lignin) concentration per unit mass. Although phosphorus, magnesium and potassium are important in several processes in plant growth and development, their role in *C*. *odorata* plants and the reason why they seasonally or phenologically and spatially vary in *C*. *odorata* leaves are unknown. The above results are consistent with our prediction that seasonality and light condition can alter the leaf characteristics of *C*. *odorata* plants. The changes in the plant characteristics due to the single, combined or interactive effects of both factors can influence the preference and performance of insect herbivores–and perhaps advance our fundamental understanding of insect plant interactions.

In this study, we observed that seasonal (or phenological) and light mediated changes in the phenotypic and phytochemical properties of *C*. *odorata* singly or interactively influenced the performance of *P*. *insulata*. For instance, development was faster, growth rate was higher, pupal mass was heavier and increased number eggs was noticeably evident in individuals that fed on shaded (relative to full-sun) or autumn (relative to winter) foliage. However neonate larvae did not display a preference for either shaded or full-sun foliage in this study. Pupal mass and development time are the most frequently used indicators that show how insects respond to their host’s nutritional quality and perhaps may predict changes to other important aspects of insect biology such as survival, behaviour, physiology and fecundity [49, 50]. A reduction in larval or pupal weight is generally known to affect future reproductive success by reducing adult body size, and possibly fecundity [34, 51]. Prolonging development time negatively affects the survival of immature stages (in a field situation) by exposing them to possible predation, parasitism, or unfavourable environmental conditions for a longer period [49, 52].

The spatial and temporal variation in the leaf characteristics of the plants can help explain the apparent superiority of shaded and autumn foliage. For instance foliar nitrogen was 69.2 and 52.5% higher in shaded plants (relative to full-sun) in both winter and autumn respectively. Increased foliar nitrogen has been linked to faster development time, higher survival rate, increased body mass, high growth rates and increased fecundity and population density in erebid moths and other insect species [1, 8, 53]. The prolonged development time, decreased pupal mass and growth rate as well as the reduced number of eggs in individuals that fed on shaded leaves in winter (when foliar nitrogen was 18% higher than autumn) relative to those that fed on shaded leaves in autumn, indicates that increased nitrogen levels in winter might have imposed a constraint on herbivore physiology. This finding supports theoretical predictions that insect herbivore performance increases with nutrient levels, but then plateaus due to diminishing returns or even declines at higher nutrient levels due to nutrient toxicity [4, 54]. The increased water content in shaded (relative to full-sun) and autumn (relative to winter) leaves as well as the reduced leaf toughness of shaded foliage in both seasons might have contributed to the faster development time, higher growth rate and increased pupal mass in *P*. *insulata*, as decreased leaf toughness and increased leaf water content are often linked to improved herbivore performance [18], although other authors [11, 12] have reported equivocal patterns. The better performance of *P*. *insulata* in autumn, correlated with a slight increase in foliar phosphorus content. The importance of dietary phosphorus on the survival, growth and development of some insects have been documented [53], but its relevance in the performance of *P*. *insulata* remains a question that needs to be investigated further. An increase in potassium and a decrease in magnesium in autumn coincided with improved performance of *P*. *insulata* in this study. Although very little is known about how variations in potassium and magnesium concentrations can affect insect herbivores, the concentrations of both elements are known to be positively associated with insect population density [55]. The lack of significant differences in pupal mass and number of eggs between individuals that fed on shaded and full-sun leaves in autumn suggests that leaf nutrients were more suitable for the herbivore than in winter. The improved performance of *P*. *insulata* on shaded foliage in both seasons supports the predictions of the C/NB hypothesis. However, the better performance of individuals of the moth on full-sun foliage in autumn compared to their counterparts that were reared on shaded or full-sun foliage in winter does not appear to extinguish the debates surrounding the C/NB hypothesis in terms of herbivore performance.

The better performance of the moth on full-sun leaves in autumn relative to full-sun or shade leaves in winter suggests that full-sun foliage growing in autumn are a suitable source of food in the absence of shaded plants. This is surprising because many studies often report improved performance of insect herbivores on shaded foliage [15, 18]. If we had not conducted studies across a seasonal spectrum (autumn vs winter), we wouldn’t have been able to detect this. The improved overall performance of the moth on autumn foliage (relative to winter) further suggests that autumn foliage satisfied the nutritional requirements of the insect possibly due to increased rainfall and or temperature during this period. Even though the population remained low in the field, our study allows us to hypothesize that *P*. *insulata* should be more abundant during autumn months because of the better quality of leaves during this period. Our results further suggest that *C*. *odorata* growing in shaded habitats or in full-sun habitats in autumn, may enhance the establishment and/or performance of biological control agents such as *Pareuchaetes* species.

Outside the indirect effects of seasonality (low winter temperature: through host plant phenology and quality) on herbivore performance (in this study), recent studies suggest that direct and indirect effects of low winter temperatures can reduce the mobility, prolong development time and compromise other life history events or traits in *P*. *insulata* [56, 57]. A combination of both direct and indirect effects of low winter temperatures (through direct effects and through host plant quality) may represent a double-blow for the insect during winter months, or in cold areas, and this may subsequently be detrimental for the populations of this insect in the field, particularly given its native-range climate.

Whatever specific nutritional attributes ultimately determine plant quality per se in this study, is far from being the only factor affecting insect fitness. The importance of, for instance, secondary chemicals (see reviews in [58, 59]) and natural enemies [60] has been proven beyond doubt, and to complicate matters further, these factors may interact with plant quality as determinants of insect herbivore performance and population density. The concentration of the secondary chemical, pyrrolizidine alkaloids (PAs) in other arctiine moths such as *Utetheisa ornatrix* (L.) (Lepidoptera: Erebidae) is known to play a key role in the mating biology and in the defence of immature stages against predators and parasitoids [58, 61]. For example, smaller adult males have less chance of mating [62], and if mating does occur, parent females may produce small-sized offspring resulting in reduced adult fecundity, and compromising the fitness of immature stages–as smaller-sized adults are likely to produce eggs with reduced PAs [58]. Whether secondary chemicals (including PAs) in *C*. *odorata* vary with seasonality, plant phenology and light intensity, and whether these variations would influence *P*. *insulata* performance still remains a question. While herbivorous insects from families with exposed larvae (such as *P*. *insulata*) are unlikely to be heavily parasitized in a new geographical range [60, 63], the role of predators on the different life stages of *P*. *insulata* needs further investigation across a spatio-temporal spectrum.

To conclude, this study demonstrated that seasonality (through its effect on host plant phenology) and light environment contributes to altered leaf chemistry (e.g. nitrogen, magnesium, phosphorus and TNC), leaf physiology (leaf water content) and leaf defence (leaf toughness) of *C*. *odorata* plants and that these variability consequently influenced the developmental and reproductive performance of *P*. *insulata* in ways that are not straightforward. These findings are largely consistent with our earlier predictions (see introduction). To our knowledge, the current study is the first to report how seasonal and light mediated variability in host plant influence herbivore performance in the bitrophic interaction between *C*. *odorata* and any arctiine moth, but whether or not it extends to other species in the broader field of light environment-effects on herbivore-host plant interactions is unknown, and requires further studies on other systems.
